# Spinal extradural meningioma mimicking lumbar disc herniation in a cat: a case report

**DOI:** 10.1186/s12917-026-05310-4

**Published:** 2026-02-13

**Authors:** Seoyeoun Ji, Hyung-Kyu Chae, Yeon-Jung Hong

**Affiliations:** 1 Department of Veterinary Radiology, Western Referral Animal Medical Center, Seoul, 04101 Republic of Korea; 2 College of Veterinary Medicine and the Research Institute for Veterinary Science, Seoul National University, Seoul, 08826 Republic of Korea; 3 Department of Veterinary Clinical Nutrition, Kyungpook National University, Daegu, 41566 Republic of Korea; 4 Korea Society of Feline Medicine, Seoul, 05556 Republic of Korea; 5 Department of Veterinary Surgery, Western Referral Animal Medical Center, Seoul, 04101 Republic of Korea

**Keywords:** Feline disease, Spinal neoplasia, Extradural meningioma, Fibrous meningioma

## Abstract

**Background:**

Neoplasia affecting the feline spinal column is an uncommon clinical occurrence, with the most frequently documented types being lymphosarcoma, osteosarcoma, glial tumors, and meningioma. This report describes the first case of an extradural meningioma affecting the lumbar spinal cord of a cat.

**Case presentation:**

An 8-year-old spayed female domestic shorthair cat presented with a two-day history of paresis, dyschezia, and reduced tail movement. Hematologic testing and thoracic radiographs revealed no abnormalities; however, lumbar radiography identified a radiopaque mass in the spinal canal at the L5-6 level. Magnetic resonance imaging demonstrated an extruded nucleus pulposus-like extradural mass with limited parenchymal enhancement, compressing the spinal cord at L5-6. A hemilaminectomy was performed, and an extradural soft tissue mass not attached to the dura mater was excised. Histopathology confirmed the diagnosis of a fibrous meningioma.

**Conclusions:**

While spinal meningiomas typically present as intradural extramedullary lesions, their occurrence in the extradural space is exceptionally uncommon, especially when no dural connection is present. This case provides the first imaging description of an extradural fibrous meningioma without dural involvement in a cat. Clinically, this highlights the importance of considering atypical forms of meningioma in the differential diagnosis of extradural spinal masses with minimal contrast enhancement.

## Background

Spinal tumors are rare in cats, with lymphosarcomas, osteosarcomas, glial tumors, and meningioma being most commonly reported [1]. While feline spinal meningiomas predominantly affect geriatric patients over 10 years of age, cases have been documented in cats as young as 3 years [1–4]. Furthermore, current veterinary literature indicates no specific predisposition regarding breed or sex [1, 2, 5].

Regarding anatomical distribution, the thoracic spine is the most prevalent site for feline spinal meningiomas, with the cervical and lumbar regions being less frequently involved [2, 6]. The neurological manifestations of feline spinal meningiomas typically reflect the severity of spinal cord compression, presenting a clinical spectrum that spans from mild spinal pain to complete paralysis [1]. The onset and severity of the neurological signs depend on the tumour’s growth rate, location, and degree of the spinal cord compression Several factors, including the rate of tumour growth, its specific anatomical site, and the extent of spinal cord compression, dictate the timing and intensity of neurological symptoms [1, 5].

Spinal meningiomas in humans are usually located in the intradural extramedullary space, and since they originate from the meninges, they are typically attached to the dura mater [7, 8]. In some cases, they are also associated with extradural extensions. However, extradural spinal meningiomas have not been reported in veterinary medicine and are rare in humans [9]. This case report describes the first case of an extradural meningioma in the lumbar spinal cord of a cat.

## Case presentation

An 8-year-old spayed female domestic shorthair cat was presented with a two-day history of paraparesis, dyschezia, and reduced tail movement.

**Fig. 1:**
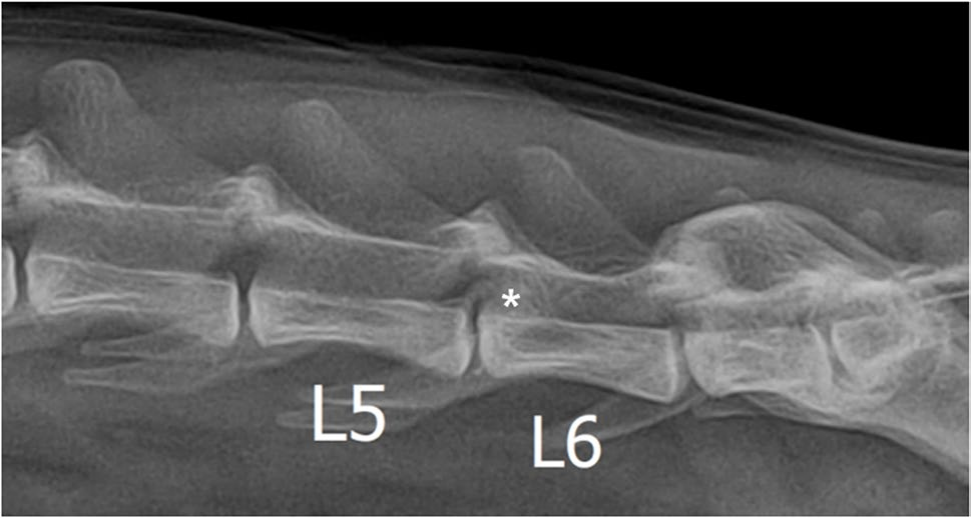
Lumbar radiographs of a cat with spinal extradural meningioma. A radiopaque area (asterisk) is identified on the dorsal side of the L5-6, which was considered a spinal compression lesion

Neurological examination revealed hind limb proprioceptive ataxia, ambulatory paraparesis of motor Grade II in both hind limbs, and decreased patella and withdrawal reflexes consistent with a lower motor neuron lesion. Hematologic testing and thoracic radiographs revealed no abnormalities. Lateral lumbar radiograph identified a radiopaque mass in the spinal canal at the L5-6 level on lumbar radiography (Fig. 1).

**Fig. 2:**
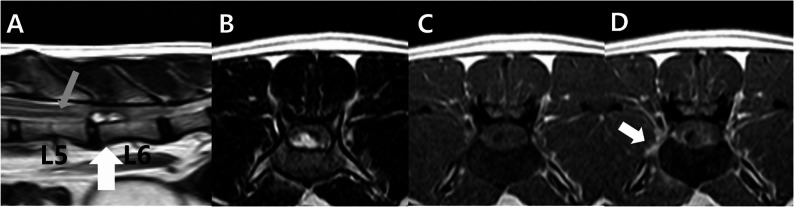
MRI of a cat with a spinal extradural meningioma. The large white arrow on the sagittal plane image denotes the position of the transverse slices. **A **A well-defined, oval-shaped mass approximately 11 × 3.8 mm in diameter in the epidural ventral space from the L5-6 to the middle of the L6 vertebral body. Compared to the spinal cord, the mass is (**B**) hyperintense in the T2-weighted image and (**C**) hypointense in the T1-weighted image. **D** After administration of contrast medium, the mass demonstrates faint and irregular contrast enhancement (white arrowhead), and paraspinal soft tissues demonstrate contrast enhancement (white arrow). **A** The mass results in spinal cord compression and T2W hyperintense changes in the spinal cord. Approximately one vertebral body length is shown. Mild syringomyelia is confirmed up to the anterior level of the mass (grey arrow). In addition, hyperintense signal decrease and slight volume reduction of the L5-6 disc nucleus pulposus are shown

A magnetic resonance imaging (MRI) was performed. General anaesthesia was induced with midazolam (0.2 mg/kg IV) and propofol (0.6 mg/kg IV) and maintained with isoflurane (0.75–1%). Magnetic resonance images of the spine were acquired using a 1.5 Tesla magnet and spine coil (Achieva Philips Healthcare, Netherlands) with appropriate repetition time (TR) and echo time (TE) parameters. The study included T2-weighted images in the sagittal and transverse directions (repetition time [TR] 3000, echo time [TE] 90, 2.5 mm), pre-and fat-saturated post-contrast T1-weighted images in the transverse and dorsal planes (TR 500, TE 9, 2.4 mm), and a gradient echo (T2*) sequence in the transverse plane (TR 324, TE 7, 2.5 mm). The post-contrast T1-weighted images were obtained after manual intravenous injection of a gadolinium-based contrast medium and gadoteric acid (Clariscan; GE Healthcare, Oslo, Norway). MRI revealed a well-defined, oval-shaped extradural mass measuring approximately 11 × 3.8 mm, extending from L5-6 to the mid-body of L6. On the T2-weighted image, the ventral epidural mass displayed a high signal intensity. At the level of the mass, the subarachnoid space was obliterated and the spinal cord was compressed (Fig. 2). The dura mater was seen as a dark line separating the extradural mass from the intradural structures. Gadolinium-enhanced T1-weighted imaging showed peripheral patchy enhancement of the mass margin, as well as regional enhancement of adjacent right-sided paraspinal muscles (quadratus lumborum and psoas major). In addition, mild syringomyelia was observed in the thoracolumbar spinal cord, located cranially to the lesion. The diagnosis was made based on the presence of a linear, continuous, and well-demarcated T2 hyperintense and T1 hypointense signal within the central spinal cord (Fig. 2A). The L5-6 intervertebral disc exhibited decreased signal intensity and a slight volume decrease in the annulus fibrosus, indicating nucleus pulposus degeneration. Given the presence of an epidural mass with surrounding soft tissue changes, neoplastic infiltration was considered likely. However, disc herniation could not be fully excluded, as the lesion was positioned dorsally to the narrowed intervertebral disc space with an attenuated nucleus pulposus and demonstrated only minimal contrast enhancement.

**Fig. 3:**
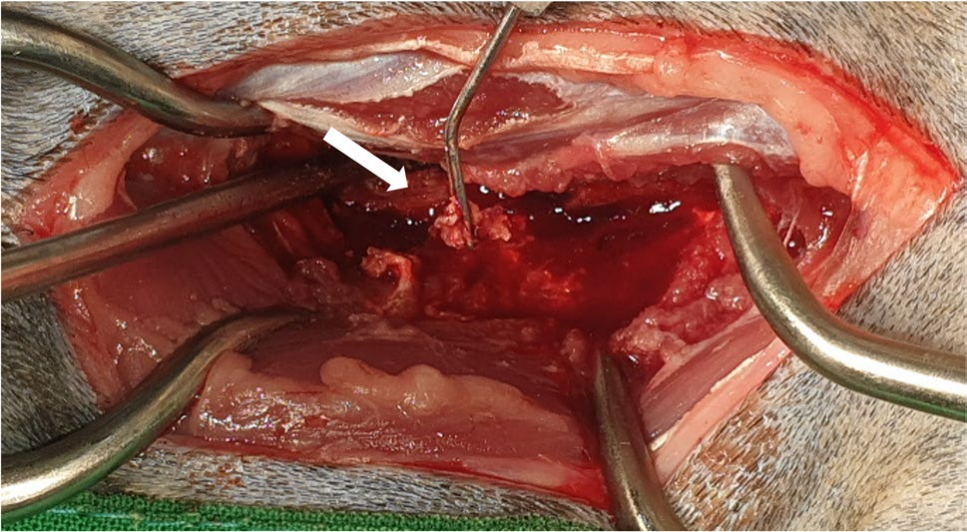
Intraoperative view of a L5-6 lumbar laminectomy. When exploring the epidural space, no dural attached or adherent point is observed around the mass lesion (white arrow). The mass is covered with a white membrane, which was dissected and opened, revealing an oval mass with bleeding

An L5-6 lumbar hemilaminectomy was performed to access the lesion. Exploration of the epidural space revealed no dural attachment or adherence to surrounding structures. The mass was encapsulated by a thin white membrane, which was incised to expose an oval, hemorrhagic lesion (Fig. 3). Associated edema of the spinal cord parenchyma and right nerve root was noted. The mass was carefully dissected and completely excised.

**Fig. 4:**
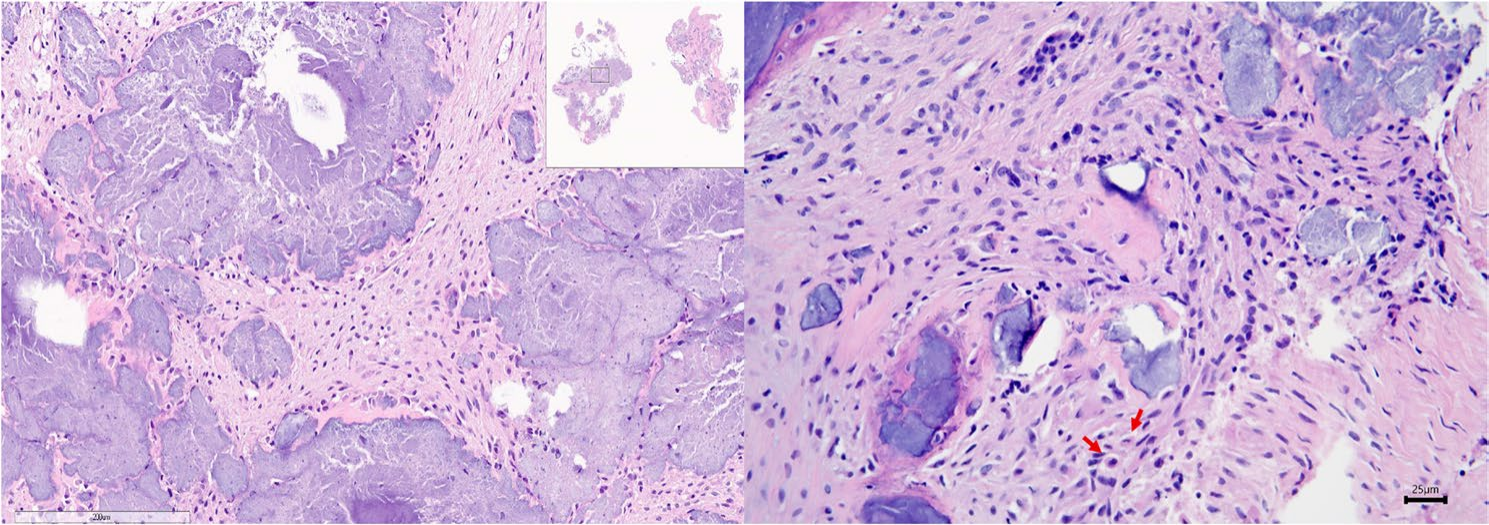
Pathologic findings of spinal extradural fibrous meningioma. The mass comprised sheets of fibrous tissue with variably sized islands of differentiated bone and cartilage. The neoplastic cells have small amounts of tapering basophilic or eosinophilic cytoplasm and oval or fusiform nuclei with stippled chromatin and mostly inconspicuous nucleoli. Mitotic figures are uncommon; three are counted in 10 high-power fields (red arrows). Scattered individual necrotic neoplastic cells are also observed (hematoxylin and eosin, × 400)

Examination of the epidural mass confirmed features consistent with a meningioma. Histologically, the mass consisted of sheets of fibrous tissue interspersed with variably sized islands of differentiated bone and cartilage. The neoplastic cells displayed scant tapering basophilic to eosinophilic cytoplasm, with oval to fusiform nuclei, stippled chromatin, and inconspicuous nucleoli. Mitotic figures were rare, with only three observed in 10 high-power fields. Scattered necrotic neoplastic cells were also present (Fig. 4). A definitive diagnosis of a typical fibrous meningioma (World Health Organization [WHO] grade I) was established based on these histopathological characteristics.

**Fig. 5:**
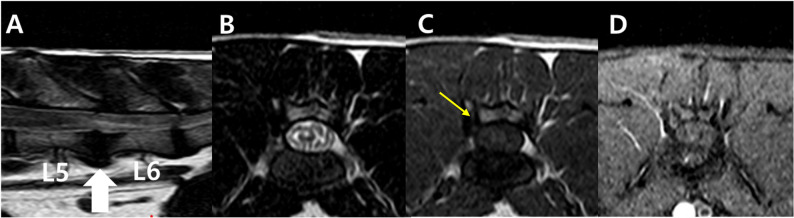
Postoperative MRI at 6 months revealed no residual mass. The large white arrow on the sagittal plane image denotes the position of the transverse slices. **A** T2-weighted sagittal image. **B** T2-weighted axial image. **C** T1-weighted axial image. A small bony defect in the right lamina of the vertebra is visible ventral to the affected articular process (yellow arrow) following right-sided hemilaminectomy. **D** T1-weighted fat saturated post gadolinium axial image

The patient’s neurological signs gradually improved postoperatively. The patient was discharged without any complications and was monitored as an outpatient. At the re-examination 6 months later, no specific neurological or MRI findings suggestive of recurrence were identified (Fig. 5).

## Discussion

Typically, spinal meningiomas arise as intradural extramedullary masses, originating from the meningothelial cells of the arachnoid membrane [7, 8]. Nevertheless, it is rarely found in extradural locations in humans due to the following hypotheses: (I) ectopic arachnoidal cell proliferation within the nerve root sleeves; (II) embryonic arachnoid remnants or villi being displaced along the dura; or (III) the translocation of arachnoid tissue clusters into the extradural compartment [10, 11].

In humans, meningiomas are histologically classified into 15 types and divided into three stages according to the WHO classification [12]. Grades 1, 2, and 3 were benign, atypical, and malignant [12]. There have been nine subtypes in dogs and cats, and various other subtypes have been reported [13]. The meningothelial, transitional, and fibrous meningioma are the most commonly reported in cats [2]. Most histopathological subtypes of non-dural-based spinal meningiomas are clear-cell meningiomas and rarely angiomatous, transitional, or meningotheliomatous subtypes in humans [14–16]. One case of nondural fibrous meningioma has been reported in humans [17]. The fibrous subtype is classified as WHO grade 1 and is characterised by benign tumours.

Due to its ability to delineate the boundaries of a lesion and assist in preoperative mapping, MRI remains the diagnostic gold standard for spinal meningiomas. Nevertheless, the rarity of extradural variants within the spinal column often leads to potential diagnostic pitfalls or clinical misinterpretation. Consequently, clinicians must approach the diagnosis of extradural spinal masses with meticulous care, as it directly dictates the surgical approach and subsequent patient prognosis. Given the atypical features of our case, specifically the poor or minimal contrast enhancement and the mass morphology that mimicked intervertebral disc material, the differential diagnosis was particularly challenging. Differential diagnosis of extradural spinal neoplasia is critical and relies heavily on key MRI features, including the degree of bone lysis and the pattern of contrast enhancement [18]. Typically, feline lymphoma—the most prevalent extradural neoplasm in the species—is characterized by its homogeneous contrast uptake, the maintenance of vertebral integrity, and a tendency for the lesion to be centered within the bone [18]. Conversely, mesenchymal tumors and primary osseous malignancies, such as fibrosarcoma and osteosarcoma, frequently manifest with distinct cortical osteolysis and non-uniform patterns of enhancement [18]. Our case’s unusual lack of strong enhancement, therefore, distinguishes it from typical lymphomas and many other highly vascularized tumors, highlighting the importance of considering rare or atypical forms of meningioma in the differential diagnosis of extradural lesions with minimal enhancement. Classical MRI features of spinal meningiomas include sharply defined, broad-based morphology, exhibiting T2-weighted hyperintensity and slight T1-weighted hyperintensity, with robust contrast uptake following gadolinium administration [19]. In contrast, the current report identifies an epidural lesion situated specifically at the dorsal aspect of the intervertebral disc space. A decrease in high signal intensity on T2WI, a slight volume decrease in the disc’s nucleus pulposus, and insufficient contrast enhancement in the parenchyma were confirmed. Disc extrusion should be considered in such cases. However, as T2W high-signal changes and contrast enhancement were confirmed in the muscle adjacent to the mass, an epidural tumour accompanied by peripheral invasion was considered, and the possibility of lymphosarcoma, which occurs more frequently in cats, was initially considered. The enhancement of the quadratus lumborum and psoas major muscles on post-contrast T1WI warrants discussion. Preoperatively, we considered that this enhancement could be due to either inflammation and edema in the paraspinal muscles caused by the epidural mass or local invasion of the tumor. While a muscle biopsy was not performed, postoperative re-examination showed no enhancement, suggesting that inflammation was the most likely causes. Histological examination confirmed that the tumour was an extradural meningioma of a fibrous subtype. Because it is a WHO grade 1 benign tumour, the patient’s prognosis is expected to be good. No neurological abnormalities were identified on reexamination six months later, and no evidence of recurrence was found on the MRI examination.

## Conclusion

In this case report, we describe a spinal extradural meningioma without dural attachment in a cat, the first such reported case in veterinary medicine. Awareness of extradural meningiomas, though rare, may improve imaging interpretation and guide clinical decision-making. Further studies with additional cases are necessary to better characterize spinal extradural meningiomas and their diagnostic features. Despite their low incidence and atypical MRI appearance, meningiomas should be considered among the differential diagnoses for extradural spinal masses, with histopathological examination remaining essential for definitive diagnosis.

## Data Availability

The datasets used and/or analyzed during the current study are available from the corresponding author on reasonable request.

## Abbreviations

*MRI*: Magnetic resonance imaging
*WHO*: World Health Organization
*L5-6*: 5th and 6th lumbar vertebrae
*T1WI*: T1-weighted imaging
*T2WI*: T2-weighted imaging
*TR*: Repetition time
*TE*: Echo time
*IV*: Intravenous
